# Supplementary material for: Comprehensive analysis of lung cancer pathology images to discover tumor shape and boundary features that predict survival outcome
Source: Sci Rep. 2018 Jul 10;8:10393. doi: 10.1038/s41598-018-27707-4 (PMC6039531; doi:10.1038/s41598-018-27707-4)
Supplement: Supplementary file 1 — Supplementary info [file 41598_2018_27707_MOESM1_ESM.pdf]

Comprehensive analysis of lung cancer pathology images to discover tumor shape and boundary features that predict survival outcome

Shidan Wang[1, #], Alyssa Chen[1, 2, #], Lin Yang[1, 3], Ling Cai[1,4], Yang Xie[1, 5, 6], Junya Fujimoto[7], Adi Gazdar[6, 8], and Guanghua Xiao[1, 5, 6,*]

[1]Quantitative Biomedical Research Center, Department of Clinical Sciences, University of Texas Southwestern Medical Center, Dallas, Texas, 75390, USA

[2]Department of Computer Sciences, Massachusetts Institute of Technology

[3]Department of Pathology, National Cancer Center/Cancer Hospital, Chinese Academy of Medical Sciences and Peking Union Medical College, Beijing, 100021, China

[4]Children's Medical Center Research Institute at UT Southwestern Medical Center, 5323 Harry Hines Blvd, Dallas, TX, 75390 USA

[5]Department of Bioinformatics, UT Southwestern Medical Center, Dallas, Texas, USA

[6] Simmons Comprehensive Cancer Center, UT Southwestern Medical Center, Dallas, Texas, USA

[7]Department of Translational Molecular Pathology, Division of Pathology/Lab Medicine, University of Texas MD Anderson Cancer Center, Houston, TX

[8]Department of Pathology, University of Texas Southwestern Medical Center, Dallas, Texas, 75390, USA

# These authors contributed equally to this manuscript.

Correspondence to:

Guanghua Xiao, Quantitative Biomedical Research Center, University of Texas Southwestern Medical Center, Dallas, Texas, 75390, USA. Email: Guanghua Xiao@utsouthwestern.edu

Tel: +1-214-648-5178

Supplementary material

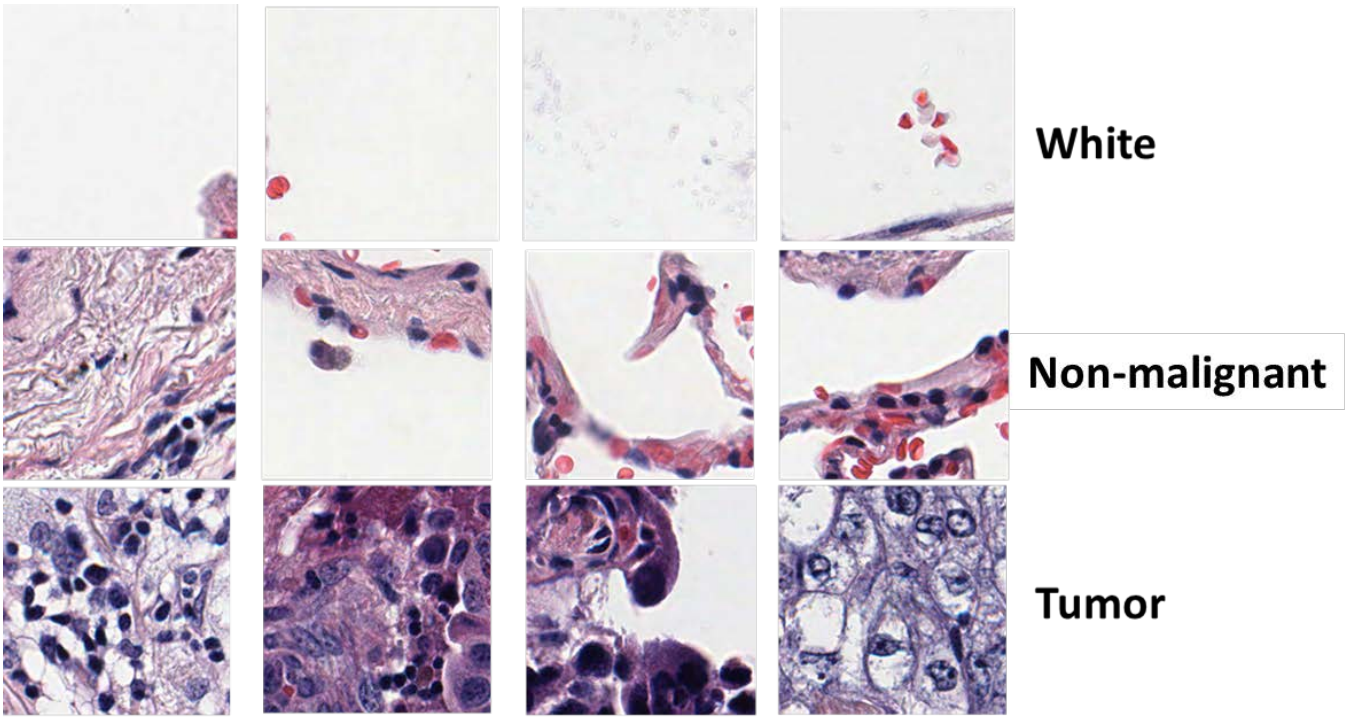

Supplemental Figure 1. Example of image patches from “white” (empty regions, upper panel), “non-malignant” (middle panel) and “tumor” (bottom panel) categories. Patch size: 300 × 300 pixels.

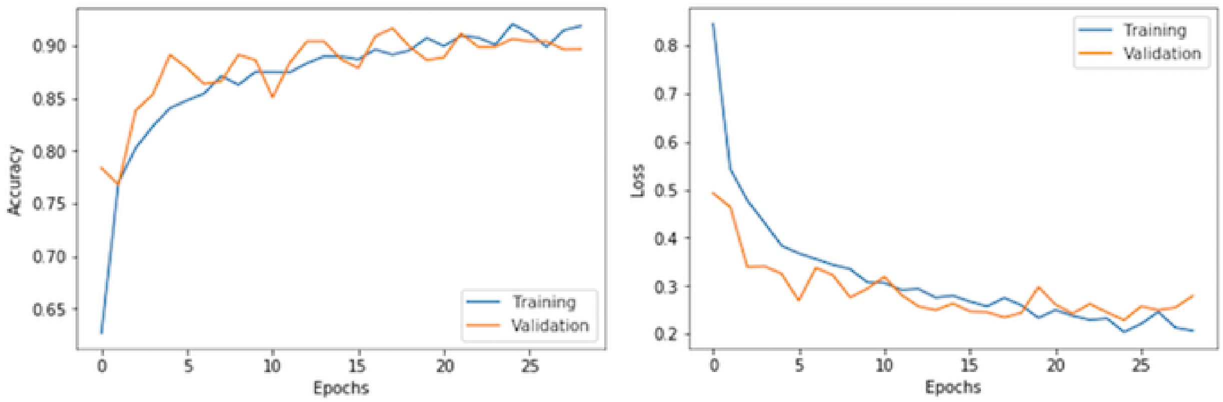

Supplemental Figure 2. Convolutional Neural Network learning curves in both training and validation datasets. Left, accuracy versus epochs; right, loss versus epochs.

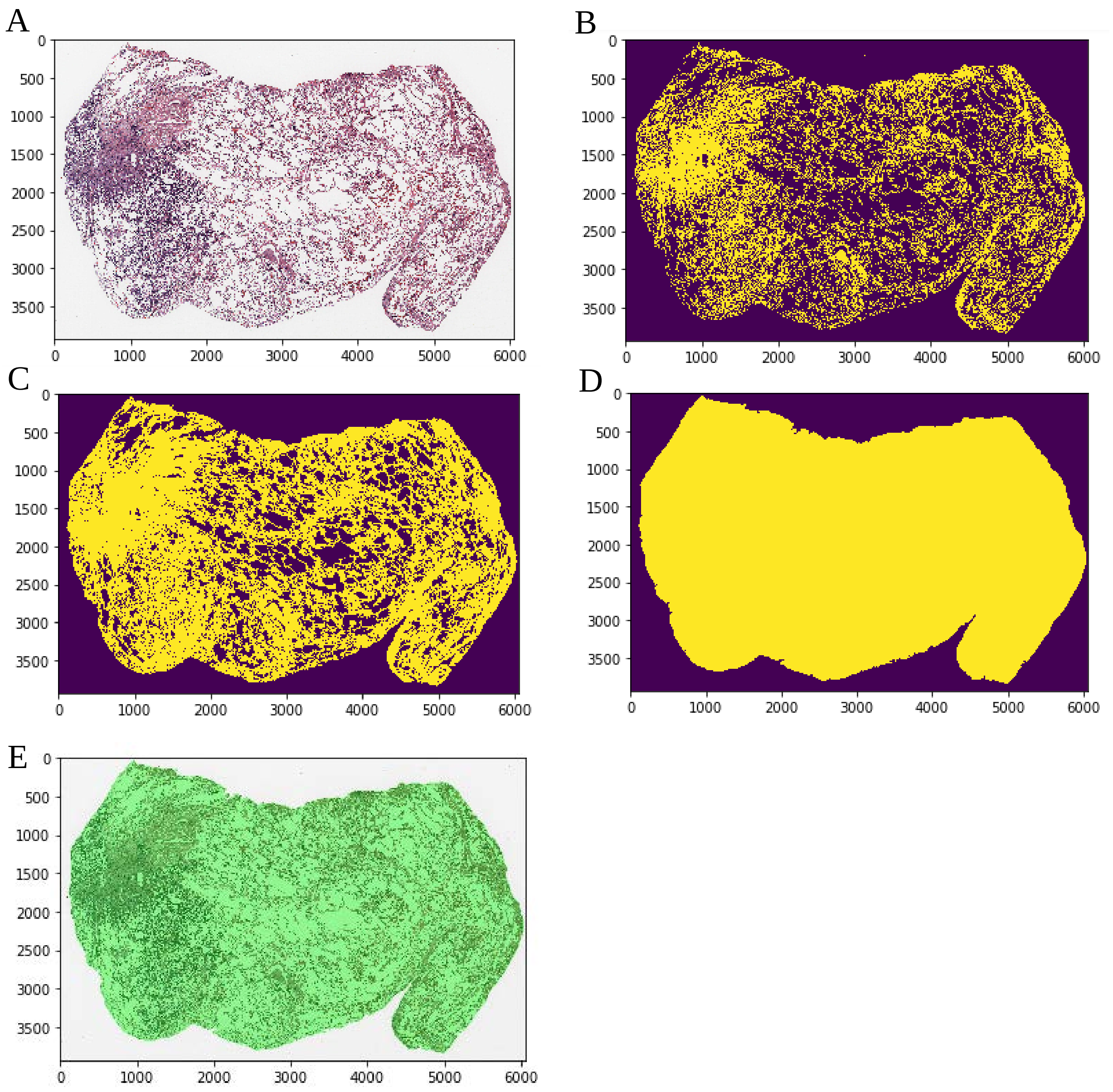

Supplemental Figure 3. Otsu thresholding and image morphological operations to speed up image-level prediction process. (A) The original image. (B) The image mask after Otsu thresholding. (C) The image mask after dilation and removal of small objects of the mask in (B). (D) The final mask after dilation, erosion, and filling up holes of mask in (C). (E) Overlap final image mask and original pathology image.

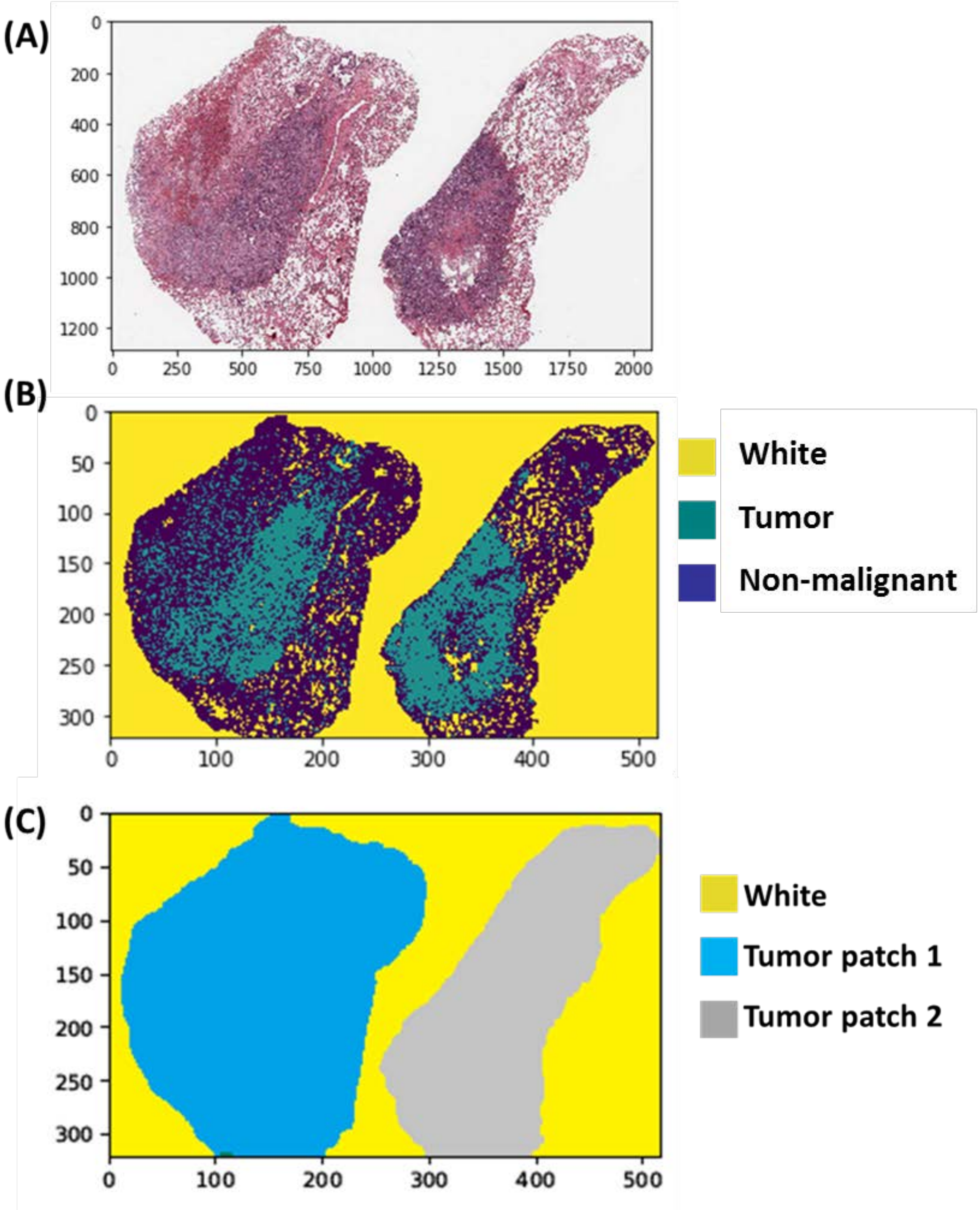

Supplemental Figure 4. Tissue region identification in case of multiple tissue samples within one image. **(A)** Original image. **(B)** Predicted patch-level tumor, non-malignant and white heatmap. **(C)** Disconnected tissue samples identified by image processing. Yellow, background; blue, first tissue patch; gray, second tissue patch.

Supplemental Table 1. Confusion matrix for image patch classification.

Ground Truth\Predicted Value	*Non-malignant*	*Tumor*	*White*
Non-malignant	400 (93.5%)	24 (5.6%)	4 (0.9%)
Tumor	58 (11.7%)	436 (88.1%)	1 (0.2%)
White	21 (14.4%)	1 (0.7%)	124 (84.9%)

Supplemental Table 2. Comparison of patient characteristics between high-risk and low-risk groups in TCGA validation dataset.

	Low-risk	*High-risk*	*p-value*
No. of patients	195	194	
Age	65.50 ± 10.11	64.45 ± 10.54	0.31
Gender			0.20
Male	81 (41.5)	94 (48.5)	
Female	114 (58.5)	100 (51.5)	
Smoking status			0.89
Yes	135 (69.2)	132 (68.0)	
No	60 (30.8)	62 (32.0)	
Stage			0.005
I	127 (65.1)	95 (49.0)	
II	44 (22.6)	52 (26.8)	
III	17 (8.7)	32 (16.5)	
IV	7 (3.6)	15 (7.7)	
